# Atomoxetine produces oxidative stress and alters mitochondrial function in human neuron-like cells

**DOI:** 10.1038/s41598-019-49609-9

**Published:** 2019-09-10

**Authors:** Juan Carlos Corona, Sonia Carreón-Trujillo, Raquel González-Pérez, Denise Gómez-Bautista, Daniela Vázquez-González, Marcela Salazar-García

**Affiliations:** 1 Laboratory of Neurosciences, Hospital Infantil de México Federico Gómez, 06720 Mexico City, Mexico; 2 Laboratorio de Investigación en Biología del Desarrollo y Teratogénesis Experimental, Hospital Infantil de México Federico Gómez, 06720 Mexico City, Mexico

**Keywords:** Cell death in the nervous system, Cellular neuroscience

## Abstract

Atomoxetine (ATX) is a non-stimulant drug used in the treatment of attention-deficit/hyperactivity disorder (ADHD) and is a selective norepinephrine reuptake inhibitor. It has been shown that ATX has additional effects beyond the inhibition of norepinephrine reuptake, affecting several signal transduction pathways and alters gene expression. Here, we study alterations in oxidative stress and mitochondrial function in human differentiated SH-SY5Y cells exposed over a range of concentrations of ATX. We found that the highest concentrations of ATX in neuron-like cells, caused cell death and an increase in cytosolic and mitochondrial reactive oxygen species, and alterations in mitochondrial mass, membrane potential and autophagy. Interestingly, the dose of 10 μM ATX increased mitochondrial mass and decreased autophagy, despite the induction of cytosolic and mitochondrial reactive oxygen species. Thus, ATX has a dual effect depending on the dose used, indicating that ATX produces additional active therapeutic effects on oxidative stress and on mitochondrial function beyond the inhibition of norepinephrine reuptake.

## Introduction

Increased hyperactivity, impulsivity and inattention are the main features of attention-deficit/hyperactivity disorder (ADHD), which is a neurobehavioral disorder in children^1,2^. The pathophysiology of ADHD is not completely understood, but has been associated with deregulation of the catecholaminergic pathway in the brain^3^, although some findings show that the pathophysiology of ADHD is associated with oxidative stress^4–6^. Regarding this, elevated levels of malondialdehyde, which is a marker of lipid peroxidation, were found in children and adults with ADHD^7–9^. Also, an oxidative imbalance was demonstrated in children and adults with ADHD^7,10^. Moreover, in a extensively accepted rat model of ADHD, spontaneously hypertensive rats (SHR) showed an increase in reactive oxygen species (ROS) production in the striatum, hippocampus and cortex^11^.

Atomoxetine (ATX) is a selective norepinephrine reuptake inhibitor and is a non-stimulant which has been approved for the treatment of ADHD due to its clinical efficacy in reducing ADHD symptoms and by increasing the quality of life^12^. ATX has gained interest as a first-line therapy for ADHD, as it has no known effect on drug abuse-related brain regions^13^. Thus, the administration of ATX increased extracellular norephinephrine and dopamine levels in the occipital cortex, cerebellum, hippocampus, prefrontal cortex and hypothalamus of rats^12,14,15^. Also, ATX binds selectively to the presynaptic norephinephrine transporter, with a little affinity for other neurotransmitters receptors or transporters^12,15^. However, there is a limited knowledge of mitochondrial alterations resulting from the therapeutic effects of ATX. Accordingly, it has been shown that ATX has additional effects beyond norepinephrine reuptake inhibition, altering gene expression and affecting several signal transduction pathways. In the SHR model, ATX up-regulates brain-derived neurotrophic factor (BDNF) expression in the prefrontal cortex, thus resulting beneficial for cognition and cellular plasticity^16^. Also, it was demonstrated that clinically relevant concentrations of ATX in cortical and hippocampal neurons from rats acted as an N-methyl-D-aspartate (NMDA) receptor blocker^17^, correlating the findings of altered glutamatergic transmission observed in ADHD^18,19^. ATX inhibits G-protein-activated inwardly rectifying K^+^ channels (GIRK), particularly brain- and cardiac-type GIRK channels, which may influence synaptic transmission and neuronal excitability^20^. Another report indicated that in the prefrontal cortex of young rats, ATX induced an increase in the expression of the ubiquinol–cytochrome c reductase complex core protein 2, as well as an increase in the GABA A receptor subunit, and in the synaptosomal-associated protein of 25 kDa, which is a membrane protein involved in synaptic vesicle exocytosis, fundamental for the synaptic transmission^21^. Recently, it was demonstrated that ATX inhibited NMDA receptors in clinically relevant micromolar concentrations and displayed voltage- and magnesium-dependent open channel blocking mechanisms in rat brain neurons^22^. The acid-sensing ion channels open in response to extracellular acidification, are widely distributed in the CNS and play a modulatory role and contribute to memory, learning processes and synaptic plasticity. Thus, ATX was demonstrated to potentiate acid-sensing ion channel response in rat brain neurons^23^.

The effects of ATX on the generation of oxidative stress and mitochondrial function have not been investigated so far. Therefore, in the present study, we evaluate whether ATX has an impact on oxidative stress and mitochondrial function in human neuroblastoma SH-SY5Y cells, differentiated into neuron-like cells.

## Results

### ATX induced cell death in human neuron-like cells

**Figure 1:**
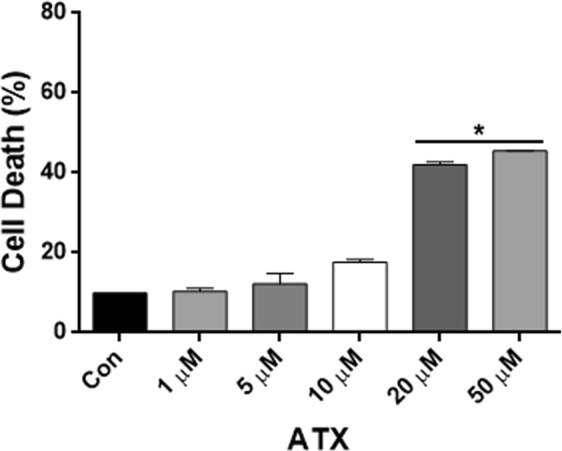
ATX induced cell death in human neuron-like cells. Cells were untreated (con) and treated with ATX for 7 days at different doses (1, 5, 10, 20 and 50 μM). Percentage of cell death at different concentrations of ATX. Data represent the mean ± SEM of three independent experiments. *P < 0.05 compared to the control group.

We carried out experiments to explore the effect of ATX over a range of concentrations on the neuron-like cells over 7 days of treatment. Figure 1 shows the percentage of cell death with a range from 1 to 50 μM of ATX. ATX exhibited a significant increase in cell death at concentrations between 20 and 50 μM (32 and 35%), while the treatment with doses 1, 5 and 10 μM produced between 1, 1.2 and 7.7% cell death, respectively, compared to control cells. In order to investigate if the cell death induced by the highest concentrations of ATX may be mediated by the generation of ROS, we analysed whether antioxidant supplementation with ascorbic acid might attenuate the effect induced by ATX. Exposure during the differentiation of cells to ascorbic acid (50 μM) alone for 7 days did not alter the cell viability (Supplementary Fig. S1). The co-treatment of cells with ascorbic acid (50 μM) and with the highest concentrations of ATX for 7 days attenuated the cell death induced by ATX (Supplementary Fig. S1).

### ATX treatment increased the rate of cytosolic and mitochondrial ROS in human neuron-like cells

**Figure 2:**
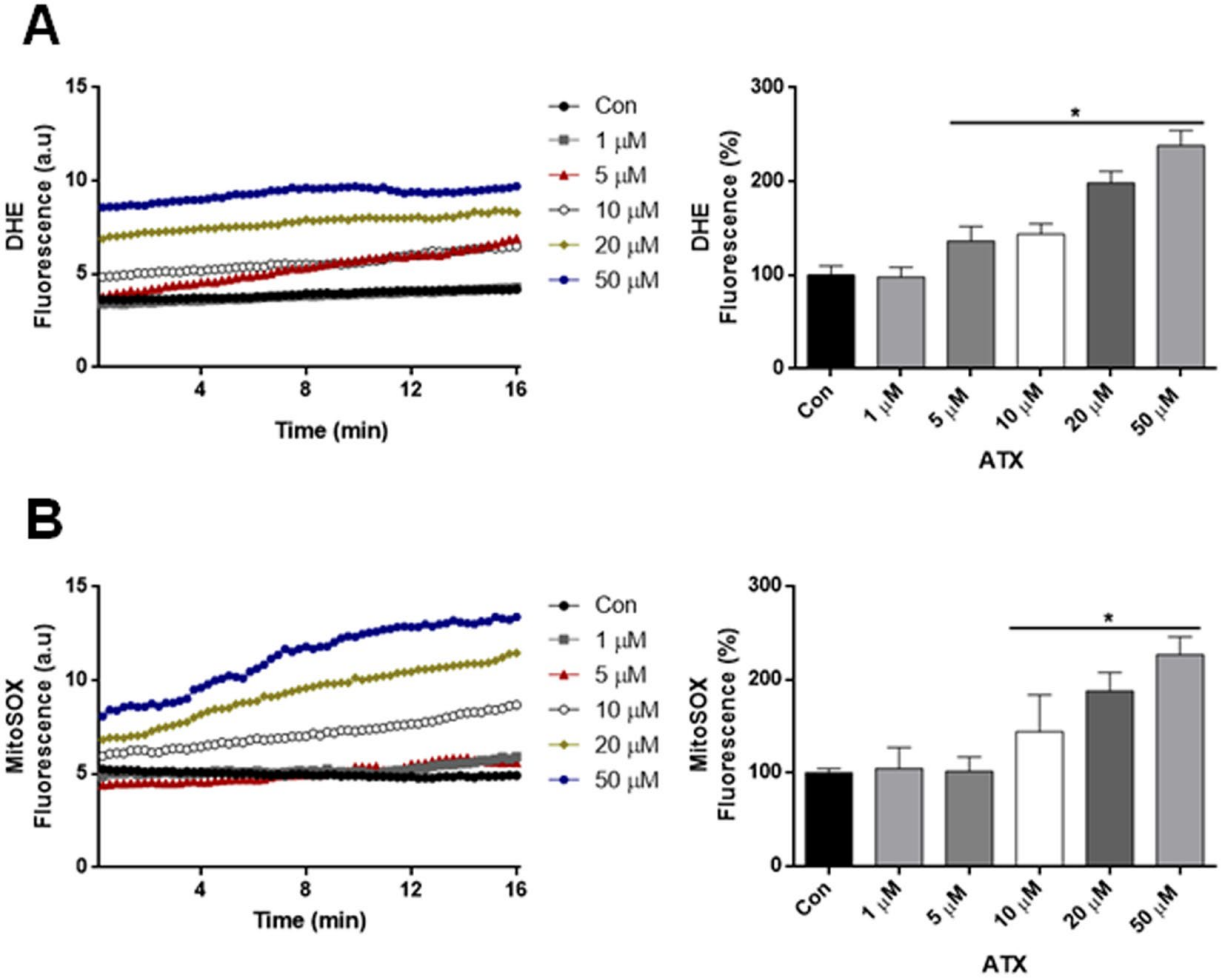
ATX treatment increased the rate of cytosolic and mitochondrial ROS in human neuron-like cells. (**A**) The increase in cytosolic ROS production in cells treated with ATX during 7 days. The quantification values of the rate of change of DHE fluorescence are shown in bar graph. (**B**) The increase in mitochondrial ROS production in cells treated with ATX. The quantification values of the rate of change of MitoSOX fluorescence are shown in a bar graph. Data represent the mean ± SEM of three independent experiments. *P < 0.05 compared to the control group.

We evaluated the effect of ATX upon the generation of cytosolic and mitochondrial ROS with the different concentrations of ATX. Cytosolic ROS production was measured with dihydroethidine (DHE). Cytosolic ROS production was increased significantly in neuron-like cells treated with ATX during 7 days in comparison with control cells (Fig. 2A), while the ROS generation was not significant with 1 μM of ATX. Similar results were observed using MitoSOX, where mitochondrial ROS production was increased significantly in neuron-like cells that were treated with 10, 20 and 50 μM; also, with the lower concentrations, there were no significant increases (Fig. 2B).

### Effects of ATX on mitochondrial membrane potential (ΔΨ_m_) and mass in human neuron-like cells

**Figure 3:**
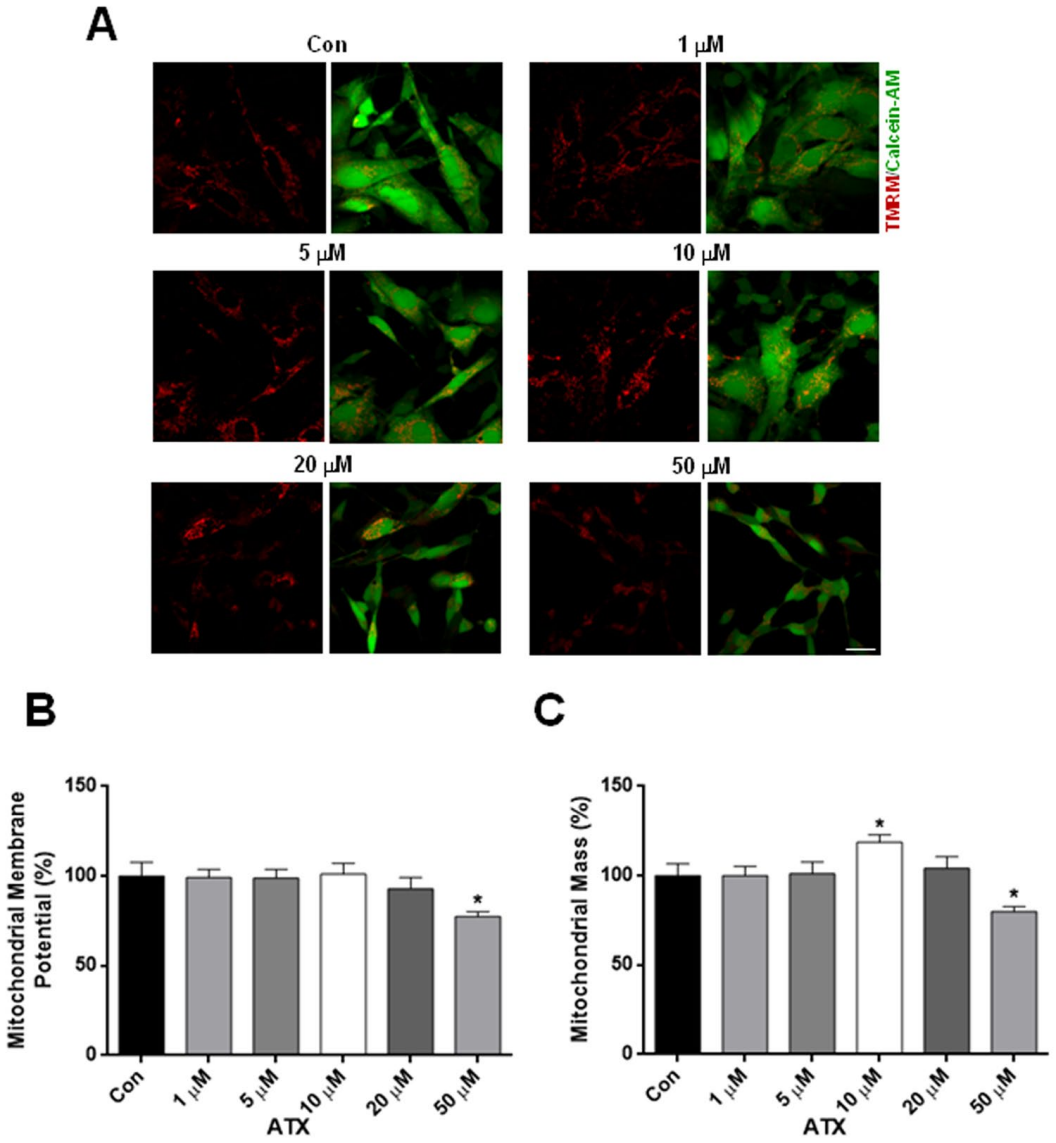
ATX altered ΔΨ_m_ and mass in human neuron-like cells. (**A**) Representative confocal images of ΔΨ_m_, were measured by the retention of TMRM (red) and mitochondrial mass which was calculated from the images using calcein-AM (green) to define the cytosol. (**B**) Quantification of ΔΨ_m_ and (**C**) mitochondrial mass. Data are mean ± SEM, and values are from five independent experiments. *P < 0.05 compared to the control group. Scale bar = 10 μm.

We further evaluated mitochondrial alterations in response to ATX treatment. Neuron-like cells were loaded with TMRM to determine changes in ΔΨ_m_. Figure 3A shows representative confocal images taken from neuron-like cells treated with the different concentrations, quantified in Fig. 3B. The treatment of neuron-like cells at 50 μM of ATX for 7 days caused a significant decrease of ΔΨ_m_ (Fig. 3B), showing that ATX induces mitochondrial dysfunction. In order to measure mitochondrial mass, neuron-like cells were co-loaded with calcein-AM to label the cytosol and with TMRM to label mitochondria (Fig. 3C). Seven days of treatment with 50 μM of ATX induced a significant decrease in mitochondrial mass, which was not observed with 1, 5, 20 μM of ATX or in control cells (Fig. 3C). Interestingly, the dose of 10 μM of ATX induced a significant increase in mitochondrial mass above that which is seen in control cells (Fig. 3C). Together these findings show that ATX has a dual effect depending on the dose used, as demonstrated by the increase in mitochondrial mass at 10 μM or by the decrease in the ΔΨ_m_ and mass with the higher concentration.

### ATX altered mitochondrial OxPhos complexes

**Figure 4:**
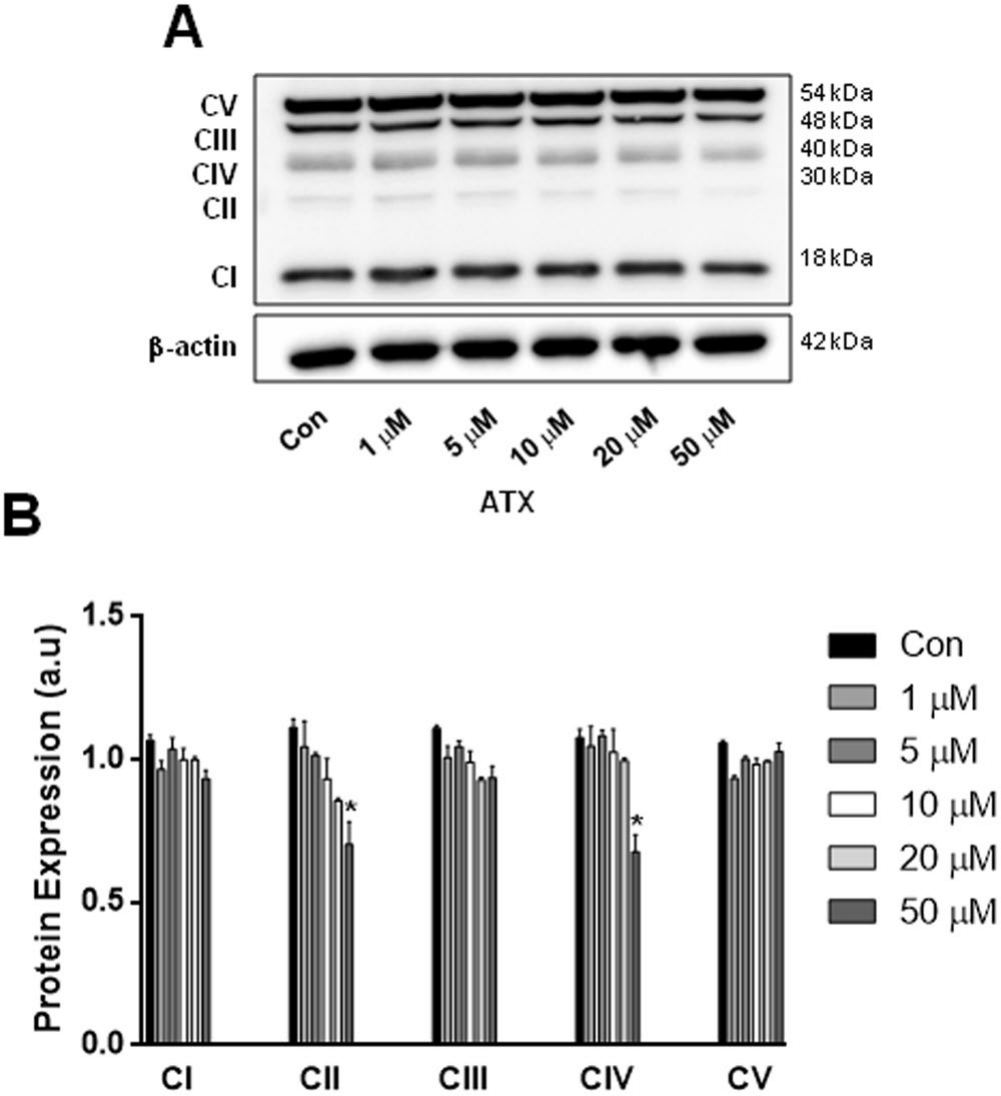
ATX altered mitochondrial OxPhos complexes. (**A**) Representative western blot. (**B**) Quantification of western blot analysis of mitochondrial OXPHOS complexes in neuron-like cells treated with different concentrations of ATX. β-actin was used as a loading control. Data are mean ± SEM, and values are from three independent experiments. *P < 0.05 compared to the control group. For clarity of the results, the representative Western blots are cropped, for raw data see Supplementary Information.

To evaluate whether mitochondrial OxPhos complexes were altered in the ATX-treated neuron-like cells, we analysed protein levels using Western blotting with a total OxPhos complex kit. Figure 4A,B revealed that ATX caused a decrease in protein contents of mitochondrial Complex II and Complex IV with 50 μM of ATX. However, there were no significant alterations in the protein contents of the other OxPhos complexes (Complexes I, III, and V), using the other concentrations of ATX when compared to control cells. This confirms that the higher concentration of ATX induced mitochondrial dysfunction.

### ATX induced alterations in autophagy in neuron-like cells

**Figure 5:**
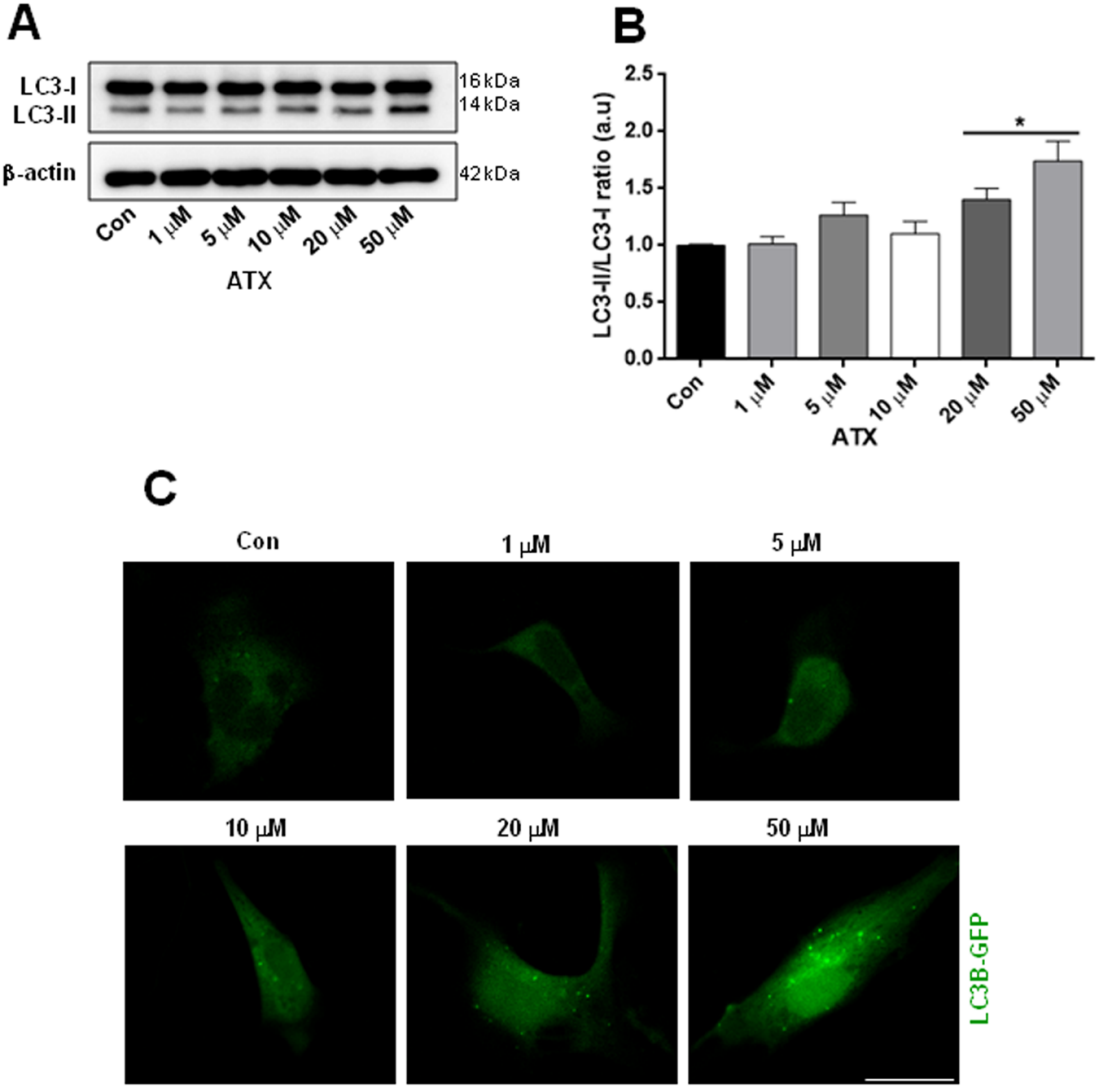
ATX induces alterations on autophagy in neuron-like cells. (**A**) Representative western blots and (**B**) quantification analysis of LC3-II levels. β-actin was used as a loading control. (**C**) Representative confocal images showed the LC3B-GFP puncta formation with different concentrations of ATX. Data are mean ± SEM, and values are from three independent experiments. *P < 0.05 compared to the control group. Scale bar = 10 μm. For clarity of the results, the representative Western blots and confocal images are cropped, for raw data see Supplementary Information.

To evaluate the role of autophagy after ATX treatment, we investigated whether autophagy is altered by the different concentrations of ATX. Autophagy was assessed using Western blot analysis of the ratio of LC3II-LC3I and to corroborate our results of western blot, we decided to use a qualitative method for which we used confocal microscopy to explore the translocation of LC3B-GFP to form puncta. Western blotting demonstrated an increased autophagy induced by the doses of 20 and 50 μM of ATX, showing a significant increase in the ratio of LC3-II/I or in the conversion of LC3-I to LC3-II (Fig. 5A,B) compared to control cells. Interestingly, the treatment of neuron-like cells with 10 μM of ATX decreased the ratio of LC3-II/I (Fig. 5B), almost back to control levels. Moreover, confocal images confirmed that the autophagosome formation was significantly increased in neuron-like cells treated with 20 and 50 μM of ATX (Fig. 5C). Taken together, these findings confirm that ATX has a dual effect, as demonstrated by the autophagy alterations, correlating the effect that ATX had on mitochondrial mass with the same doses.

## Discussion

The catecholamines (dopamine and norepinephrine) are neurotransmitters in the central nervous system: nevertheless, dopamine and norepinephrine can easily undergo auto-oxidation forming ROS^24–26^. Therefore, reaction products formed by the oxidation of catecholamines result in cell damage and damage to DNA^27,28^. It has been shown that norepinephrine can be either damaging or protective to cells, depending on the norepinephrine concentration and cell type^29–31^. Thereby, low levels of norepinephrine were shown to protect dopaminergic neurons^32^. Moreover, norepinephrine-induced endoplasmic reticulum stress and downregulation of norepinephrine transporter density in PC12 cells were via oxidative stress^33^. It was demonstrated that dopamine-induced toxicity in SH-SY5Y cells, via extracellular auto-oxidation of dopamine and consequent oxidative stress, also it was demonstrated that ascorbic acid effectively blocked the toxic effects of dopamine, suggesting that extracellular auto-oxidation of dopamine is responsible for the oxidative stress^34^. As mentioned above, ATX is a selective norepinephrine reuptake inhibitor that increases extracellular norephinephrine and dopamine levels^12,15^. The excessive accumulation of extracellular norepinephrine due to the action of a high concentration of ATX could mean that norepinephrine undergoes auto-oxidation and initiates several events such as ROS generation, which would generate cell damage, affecting mitochondrial function.

We showed that the highest concentrations of ATX increased the rate of cytosolic and mitochondrial ROS in human neuron-like cells (Fig. 2), and as a consequence produced cell death (Fig. 1). Also, we demonstrated that the co-treatment of cells with ascorbic acid plus the highest concentrations of ATX for 7 days, attenuated the cell death induced by ATX (Supplementary Fig. S1). Thus, demonstrating that ascorbic acid, which is an antioxidant is capable to protect the cells against oxidative stress, attenuating the cell death, due to its scavenger capacity with extracellular and intracellular ROS species as demonstrated previously^35,36^. This is supported by the fact that ATX increased ROS (superoxide levels) in T-lymphocytes and caused a dose-dependent decrease in cell number^37^. Also, the effects of ATX on development of the brain have been reported in rats, where ATX might have neurotoxic effects; this has been demonstrated by neurodegenerative effects on the cerebellum and hippocampus. Thus, the authors suggest that ATX neurotoxicity may arise as a result of ROS formation due to catecholamine oxidation^38^. The psychostimulant methylphenidate (MPH) is the first choice of treatment for ADHD, increasing extracellular dopamine and norepinephrine levels in the brain^39^. Hence, the acute and chronic use of MPH in adult SHR increased oxidative stress and induced energetic metabolism alterations^40^. The maintenance of ΔΨ_m_ is an important factor for cell health; therefore, a decrease in ΔΨ_m_ will result in mitochondrial dysfunction. The decrease in ΔΨ_m_ and the decrease in mitochondrial complexes II and IV were alterations found in response to ATX treatment, suggesting that ATX treatment causes a damage to mitochondrial function at the highest concentration used. Accordingly, mitochondrial defects can also lead to ROS generation^41,42^.

Disturbances in mitochondrial homeostasis may result from impaired balance in the pathways that promote mitochondrial repair (biogenesis) and pathways that remove dysfunctional mitochondria (mitophagy); the impaired coordination between both processes is a feature of several neurodegenerative disorders^43^. The observed dual or biphasic response with different doses of ATX in mitochondrial mass suggests either increased or decreased removal by autophagy. Autophagy is crucial for the maintenance of cellular homeostasis in physiological conditions because it mediates the removal of dangerous constituents such as damaged organelles, proteins and dysfunctional mitochondria; however, its role in cell death and survival remains controversial. For example, autophagy is up-regulated when cells need to generate intracellular nutrients and energy, such as starvation or high bioenergetic demands and is also up-regulated during oxidative stress, which allows cells to survive; in contrast, deregulated or insufficient autophagy can promote cell damage^44,45^. Using Western blotting and a fluorescent autophagosome marker, LC3B-GFP, we found that ATX at the highest concentrations induced an increase in autophagy in neuron-like cells, while 10 μM ATX decreased the levels of autophagy. Thus, the biphasic response to autophagy appears to be essential to the changes in mitochondrial mass associated with 10 μM ATX treatment. In that sense, it was demonstrated that ATX used at a lower dose was effective in the prevention of skeletal muscle atrophy in a model of dexamethasone-induced muscle atrophy, by sustaining PGC1α expression; PGC1α is a transcriptional co-activator that regulates mitochondrial biogenesis^46^. Also, treatment with lower doses of ATX significantly attenuated the cognitive deficits in post-traumatic injured rats^47,48^. Moreover, mitochondrial ROS generation depends on mitochondrial metabolism and may constitute the mechanism by which damaged mitochondria are selectively targeted for autophagic removal. We found that the highest concentrations of ATX increased ROS production and autophagy in neuron-like cells, but there were no such effects at lower concentrations.

Therefore, the biphasic response by ATX in neuron-like cells may be mediated by the dose and trigger of several mechanisms, including oxidative stress and autophagy. Mitophagy can be triggered by increased ROS generation^41,49,50^. Methamphetamine is a psychostimulant used for the treatment of ADHD which induces the release of dopamine from vesicles to the cytosolic and extracellular space, resulting in neurotoxicity^51^. Thus, the neurodegeneration of dopaminergic neurons induced by methamphetamine has been shown to increase autophagy^52^. Also, methamphetamine induces a decrease in ΔΨ_m_, a decrease in mtDNA copy number and an increase in ROS levels. Furthermore, the use of vitamin E attenuated cell death and an increase in intracellular ROS levels induced by methamphetamine^53^. Therefore, it has been demonstrated that enhanced antioxidant defences, ROS scavengers or the overexpression of antioxidants reduced levels of autophagy^41,49,54^.

Regarding the pharmacokinetics and the effective concentrations *in vivo*, it has been demonstrated that ATX is metabolized through the cytochrome P-450 2D6 enzyme pathway, which is polymorphic in humans, leading to a bimodal distribution of the pharmacokinetics; divided into poor metabolizers (7% Caucasians) and extensive metabolizers. In children and adolescent with ADHD, a study evaluated the pharmacokinetics of ATX, C_max_ detected ranged from 80 to 212 ng/ml with 10 mg dose^55^. A twice-daily dose 20–45 mg of ATX, C_max_ detected ranged from 174 to 1221 ng/ml, which means that the latter concentration close to 5 μM. Thus, the total plasma exposure in poor metabolizers of ATX is approximately 10-fold higher when compared with extensive metabolizers^56^. Lempp *et al*. in the prefrontal cortex of rats, adjusted the dosing procedure closely resemble the clinical paediatric treatment (oral administration, once daily, long-term application), obtained a C_max_ 139 ± 17 ng/ml, according with the data published by Witcher *et al*.^21,55^. A microdialysis study in rats demonstrated a high brain penetration of ATX concentration in brain cells higher than in plasma^57^. Thus, plasma concentrations of ATX are in the micromolar range. Besides, it was demonstrated that ATX blocked NMDA receptor in the micromolar range^17^.

Finally, as mentioned before, it has been shown that ATX has additional effects, affecting several signal transduction pathways and altering gene expression^16,17,20,21^; for that reason, it is necessary to add the effects on the oxidative stress and on mitochondrial function as demonstrated here.

In conclusion, our findings suggest that treatment with the highest concentration of ATX in neuron-like cells caused mitochondrial alterations, enhanced oxidative stress, disturbed mitochondrial mass and autophagy, indicating that ATX activated these events in a consecutive manner. The enhanced oxidative stress might trigger alterations in ΔΨ_m_ and aberrant mitochondrial biogenesis, which results in a decrease in mitochondrial mass and an increase in autophagy after ATX treatment. Dysfunctions in the mitochondria are a source of ROS; therefore, mitochondrial alterations might provide a sequence of mechanisms by which ATX induces more oxidative stress and increases cell death. As a consequence, additional studies are required to characterise the dose of ATX and to determine a window of time and concentration in which the ATX, beyond being harmful, can be beneficial, especially for patients who use this drug for the treatment of ADHD. This is because we found that 10 μM of ATX had a positive effect, as demonstrated by the increase in mitochondrial mass and the decrease in autophagy, at least in our conditions and in our cell model. Therefore, ATX has a biphasic response depending on the dose used, indicating that ATX produces additional active therapeutic effects on oxidative stress and on mitochondrial function beyond the inhibition of norepinephrine reuptake.

## Methods

### Reagents and antibodies

DMEM, F12, foetal bovine serum (FBS), penicillin-streptomycin, total OxPhos complex kit, Tetramethylrhodaminemethyl ester (TMRM), calcein-AM, Premo™ Autophagy Sensor LC3B-GFP, MitoSOX and Dihydroethidine (DHE) were obtained from Molecular probes-Invitrogen. Atomoxetine hydrochloride (ATX), ascorbic acid, retinoic acid, MTT and dimethyl sulphoxide (DMSO) were obtained from Sigma-Aldrich. The β-actin antibody (1:1000) and the LDH-cytotoxicity assay kit were obtained from Abcam (Cambridge, MA). The LC3B antibody was obtained from Cell Signalling Technology. Matrigel Matrix was obtained from Corning.

### Cell culture

Human neuroblastoma SH-SY5Y cells, grown in DMEM/F12 medium supplemented with 10% FBS, containing streptomycin/penicillin (100 µg/ml and 100 U/ml, respectively), in a humidified atmosphere with 37 °C and 5% CO_2_. The cells were differentiated as follows: the cells were seeded on Matrigel matrix-coated culture dishes and allowed to attach for 24 h. The cells were exposed to 10 μM retinoic acid every 2 days and the FBS content of the culture medium was then reduced to 2%. The cells were pre-treated for 7 days with ATX 1, 5, 10, 20 and 50 μM (ATX was added every 2 days at the same time that retinoic acid). After 7 days of differentiation, control cells and cells treated with ATX were used for the different determinations. We selected the doses of ATX according to the reported clinically relevant concentrations in plasma, which are in the micromolar range^17,22^. *In vitro* studies are good tools in the quest to find some of the cellular and molecular mechanism of the drugs used as a therapy in ADHD. Some of the advantages of using *in vitro* the human differentiated SH-SY5Y cells are: differentiated cells possess more morphological, ultrastructural, biochemical, and electrophysiological similitude to neurons. The cells present the formation of synaptophysin-positive functional synapses, and induction of neuron-specific enzymes, neurotransmitters, and neurotransmitter receptors. The differentiated cells could express the norepinephrine transporter and the vesicular monoamine transporter, characteristic of adrenergic neurons. Besides, the differentiated cells have many characteristics of dopaminergic neurons, since are positive for tyrosine hydroxylase and dopamine-β-hydroxylase, as well as express the dopamine transporter^58,59^.

### Cytotoxicity assay

Lactate dehydrogenase (LDH) cytotoxicity assay was performed in differentiated cells treated with different concentrations of ATX using an LDH cytotoxicity assay kit, according to the manufacturer´s instructions (Abcam, Cambridge, MA). Briefly, cells were seeded and differentiated on white, clear-bottom 96-well plates at a density of 2 × 10^6^ cells per well. Five microliters of media from each well was then mixed with 95 μL of the reaction mixture (supplied in the kit), followed by the measurement of fluorescence at excitation and emission wavelengths of 535 nm and 587 nm, respectively. Each experiment was repeated three times using separate cultures.

### MTT assay

Cell viability was determined using MTT assay (Sigma, Saint Louis MO, USA) in differentiated cells treated with the highest concentrations of ATX or with ascorbic acid (50 μM). Cells were seeded and differentiated on plates at 37 °C in a humidified atmosphere and 5% CO_2_. The medium was removed, cells were washed and 100 μl of MTT of stock (5 mg/ml) in PBS was added to the cultures, after 4 h of incubation, the solution was removed and 100 μl of isopropanol was added to dissolve the resulting formazan salts. Following 5 min, the wells were read at 540 nm on an spectrophotometer. Each experiment was repeated three times using the same experimental conditions. The results were expressed as percentage.

### Measurement of mitochondrial mass and mitochondrial membrane potential (ΔΨ_m_)

Once differentiated on coverslips for 7 days, cells were loaded with Hank’s Balanced Salt Solution (pH 7.4), containing 1 μM calcein-AM and 25 nM TMRM for 30 min at room temperature (RT). Images were acquired using a Zeiss Axiovert 100M confocal microscope with a Plan-Neofluar ×63/1.25 oil immersion objective lens at RT. Calcein-AM was excited at a wavelength of 488 nm and TMRM fluorescence at 543 nm using a laser. All the images were analysed using the software Fiji ImageJ. The measurements of mitochondrial mass and ΔΨ_m_ were realised as previously reported^41^.

### Measurements of cytosolic and mitochondrial reactive oxygen species (ROS)

After differentiation, cells were loaded with Hank’s Balanced Salt Solution and dihydroethidine (DHE) (5 μM - for the measurement of cytosolic ROS) or with MitoSOX (5 μM - for the measurement of mitochondrial ROS) for 15 min and remained in solution for the duration of the experiment. Images were acquired using a Zeiss Axiovert 100M confocal microscope with a Plan-Neofluar ×63/1.25 oil immersion objective lens at RT. DHE and MitoSOX fluorescence were excited at wavelength of 543 and 488 nm respectively. The increase in red fluorescence (excited at 543 or 488 nm and measured at >560 nm with a long-pass filter) gives the rate of cytosolic and mitochondrial ROS generation. In all experiments, data were collected every 15 s for 16 min. The rate of cytosolic and mitochondrial ROS in cells treated with ATX was compared with the rate of ROS in control cells.

### Western blotting

Total proteins were extracted using standard protocols. Proteins were subjected to SDS–PAGE, polyvinylidene difluoride (PVDF) membrane (Immobilon-P, Millipore, Bedford, MA, USA) blocked for 2 h at RT with 5% non-fat dried milk in PBS, 0.2% Tween-20 (PBST). Blocked membranes were incubated overnight with primary antibodies at 4 °C. The membranes were then rinsed three times in PBST and incubated with the corresponding horseradish peroxidase-conjugated secondary antibody for 2 h at RT. Chemoluminescence signal was produced with (ECL-BioRad) and detected by Fusion-Solo WL system (Vilber Lourmat). Protein bands were quantified densitometrically with Fiji ImageJ software.

### LC3B-GFP autophagosome analysis

We used BacMam LC3B-GFP as a marker for autophagy. Once differentiated on coverslips for 7 days, control cells and cells treated with different concentrations of ATX were transfected with BacMam LC3B-GFP or BacMam LC3B (G120A)-GFP viral particles (MOI = 30) for 18–20 h, according to the Premo Autophagy Sensor Kit. LC3B-GFP and LC3B (G120A)-GFP signals were monitored and captured using a Zeiss Axiovert 100M confocal microscope.

### Statistical analysis

Statistical analysis was performed with GraphPad Prism Software (Version 6.01, Inc., La Jolla, CA). The mean ± S.E.M. values from at least three independent experiments are shown. The data were compared using one-way ANOVA with *post-hoc* Bonferroni test. Differences were considered significant when p < 0.05.

## Supplementary information

Supplementary Material
