# Supplementary material for: Atomoxetine produces oxidative stress and alters mitochondrial function in human neuron-like cells
Source: Sci Rep. 2019 Sep 10;9:13011. doi: 10.1038/s41598-019-49609-9 (PMC6737196; doi:10.1038/s41598-019-49609-9)
Supplement: Supplementary file 1 — Supplementary Material [file 41598_2019_49609_MOESM1_ESM.docx]

**Atomoxetine produces oxidative stress and alters mitochondrial function in human neuron-like cells**

Juan Carlos Corona^1,^*, Sonia Carreón-Trujillo^1^, Raquel González-Pérez^1^, Denise Gómez-Bautista^1^, Daniela Vázquez-González^1^ and Marcela Salazar-García^2^.

^1^Laboratory of Neurosciences, Hospital Infantil de México Federico Gómez, 06720, Mexico City, Mexico.

^2^Laboratorio de Investigación en Biología del Desarrollo y Teratogénesis Experimental, Hospital Infantil de México Federico Gómez, 06720, Mexico City, Mexico.

*Corresponding Author: Juan Carlos Corona

E-mail: jcorona@himfg.edu.mx

**Supplementary Material**

**Figure S1**

**Supplementary Figure Legend**

**Figure S1.** MTT assay of neuron-like cells treated with highest concentrations of ATX (20 and 50 μM) and the co-treatment of cells with ascorbic acid (50 μM). There were no significant differences as compared to the control group. All the values are expressed as mean ± SEM of three independent experiments. *P < 0.01

**Full length gels in figure 4A Supplementary Figure 2**





**β-Actin**

**Con 1 μM 5 μM 10 μM 20 μM 50 μM**

**42 kDa**





**54 kDa**

**48 kDa**

**40 kDa**

**30 kDa**

**18 kDa**

**CV**

**CIII**

**CIV**

**CII**

**CI**

**Con 1 μM 5 μM 10 μM 20 μM 50 μM**

**Full length gels in figure 5A Supplementary Figure 3**

**

**

**β-Actin**

**Con 1 μM 5 μM 10 μM 20 μM 50 μM**

**42 kDa**





**LC3-I**

**LC3-II**

**16 kDa**

**14 kDa**

**Con 1 μM 5 μM 10 μM 20 μM 50 μM**

**Full length figures in figure 5C Supplementary Figure 4**


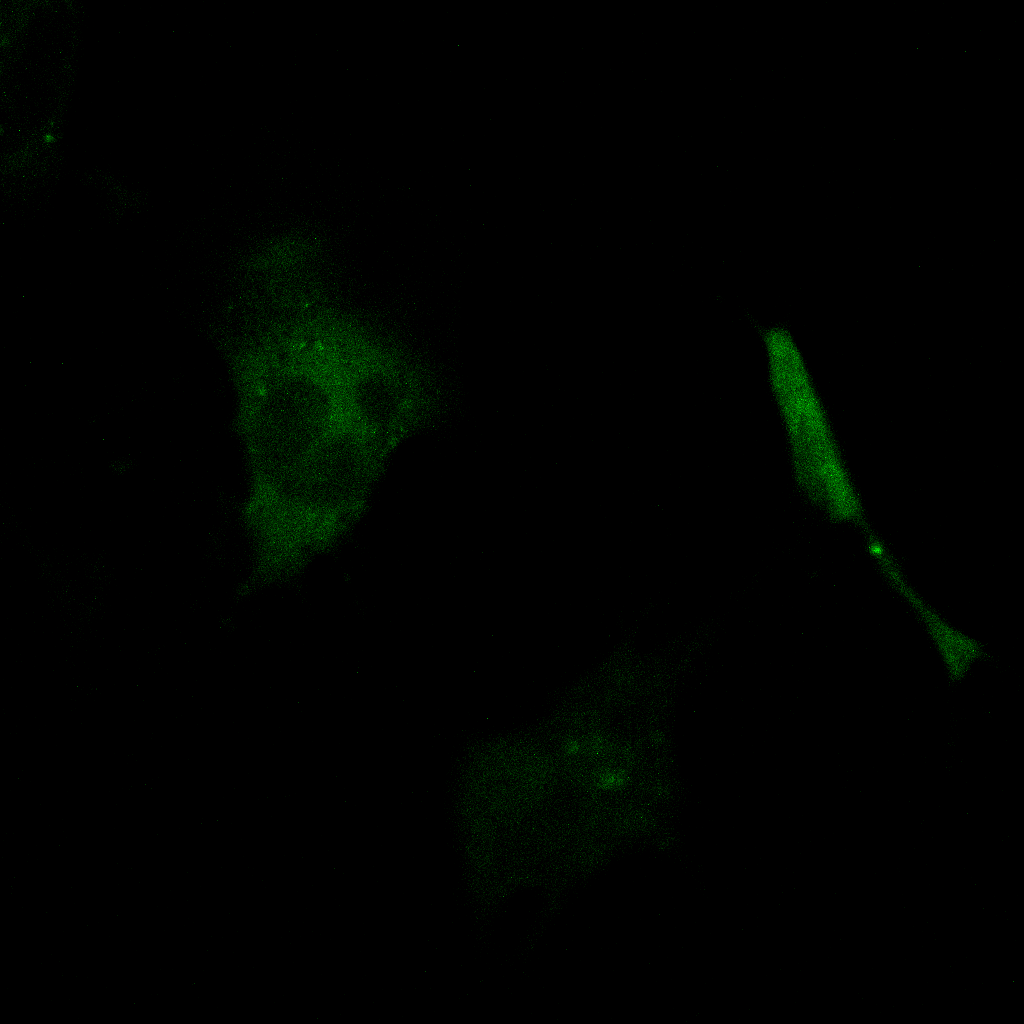

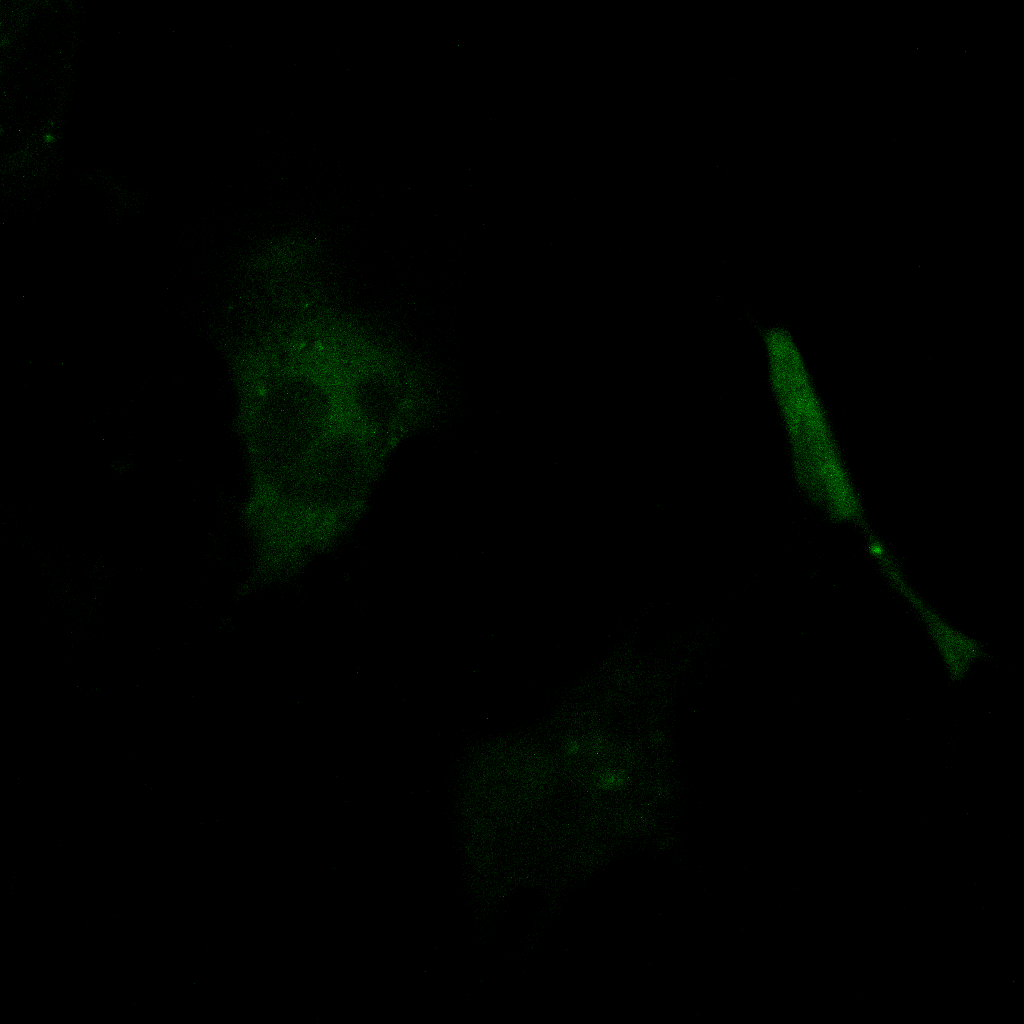


**Con**


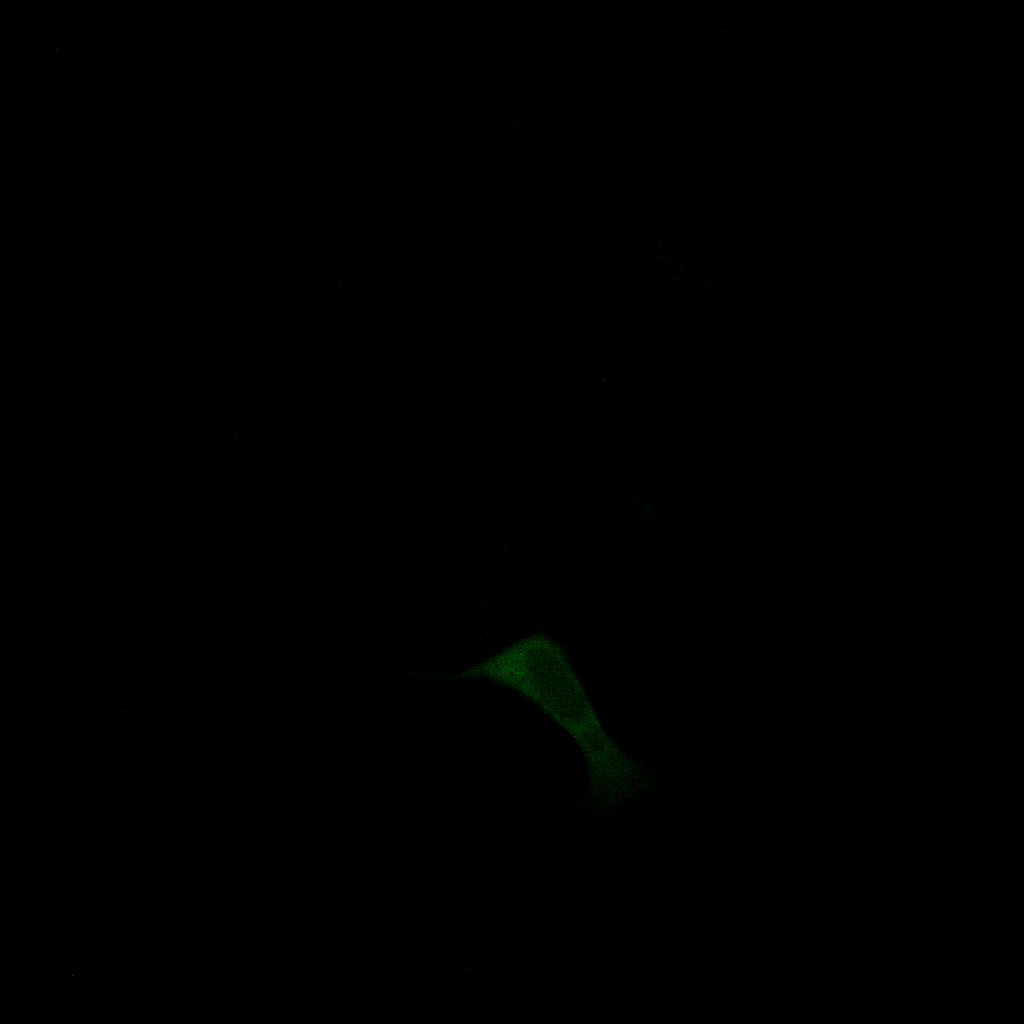


**1 μM**


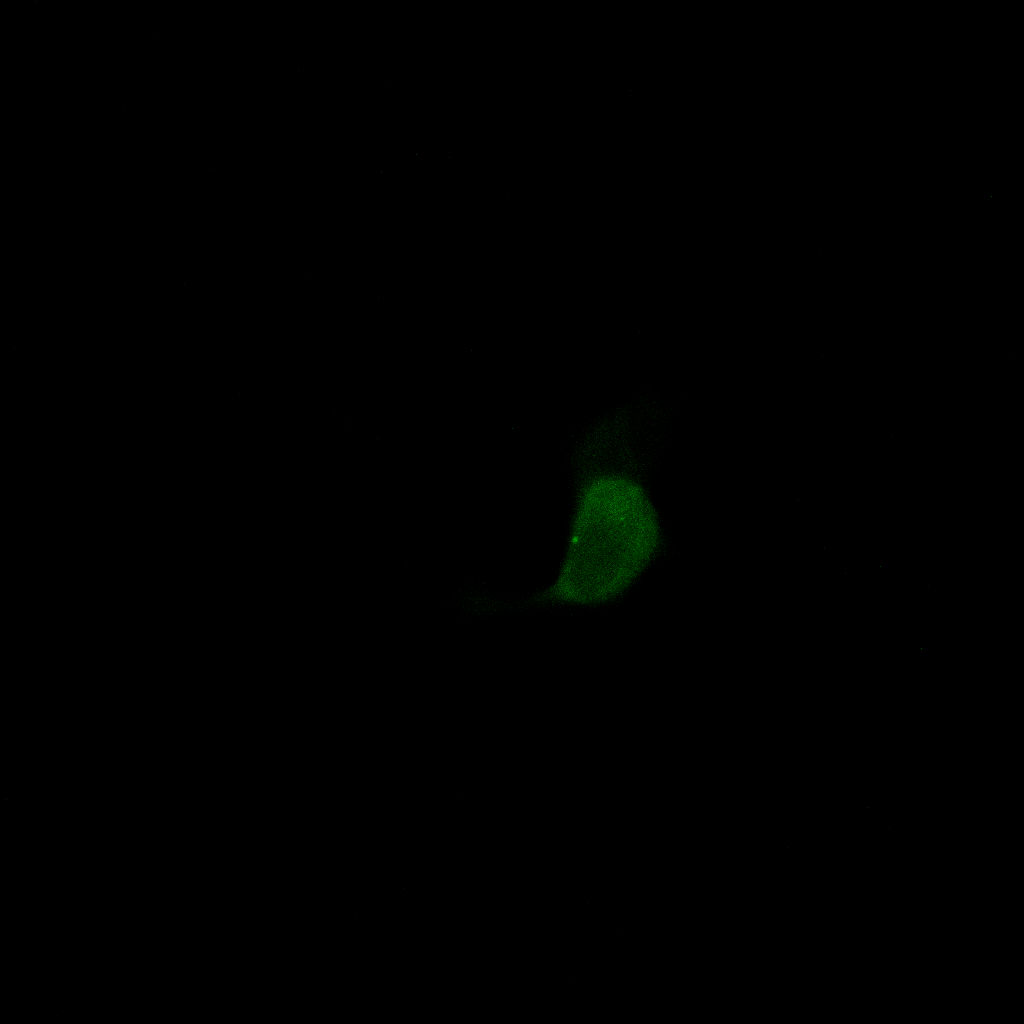


**5 μM**


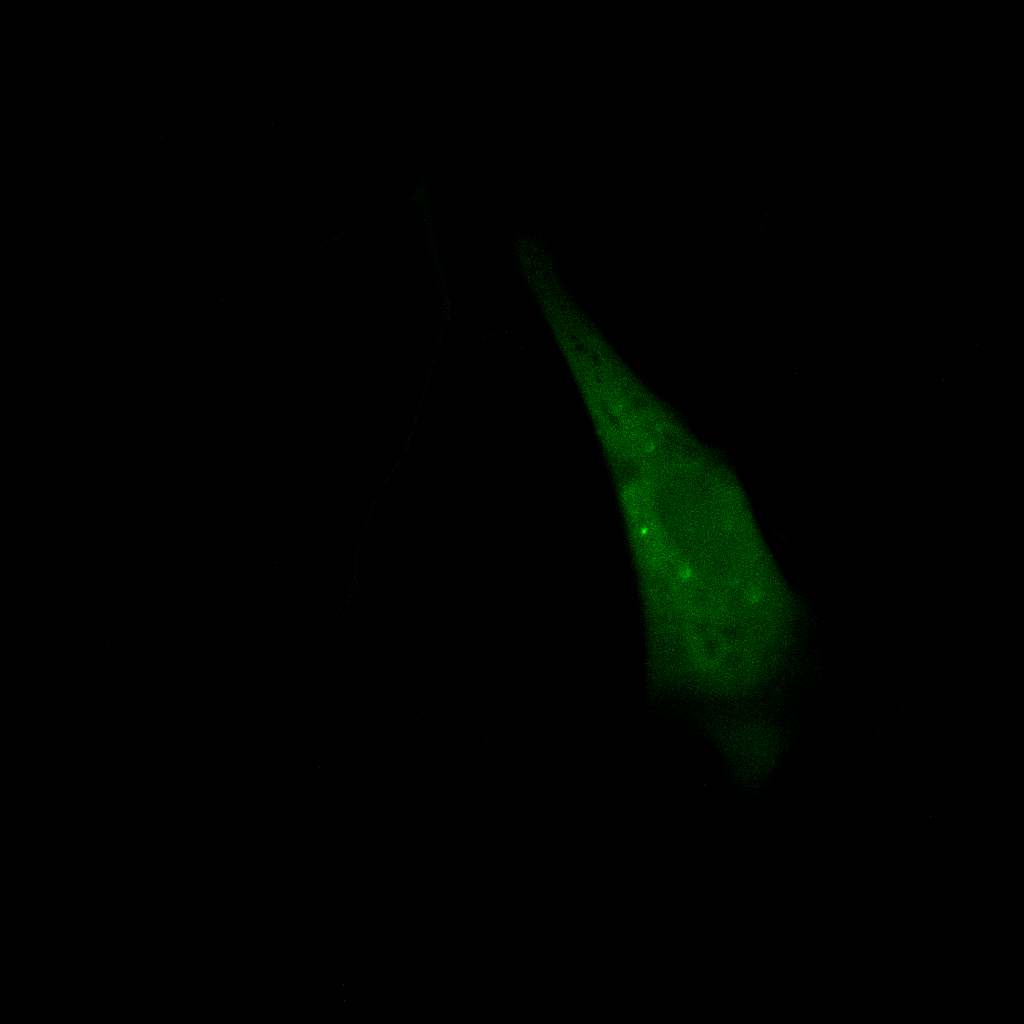

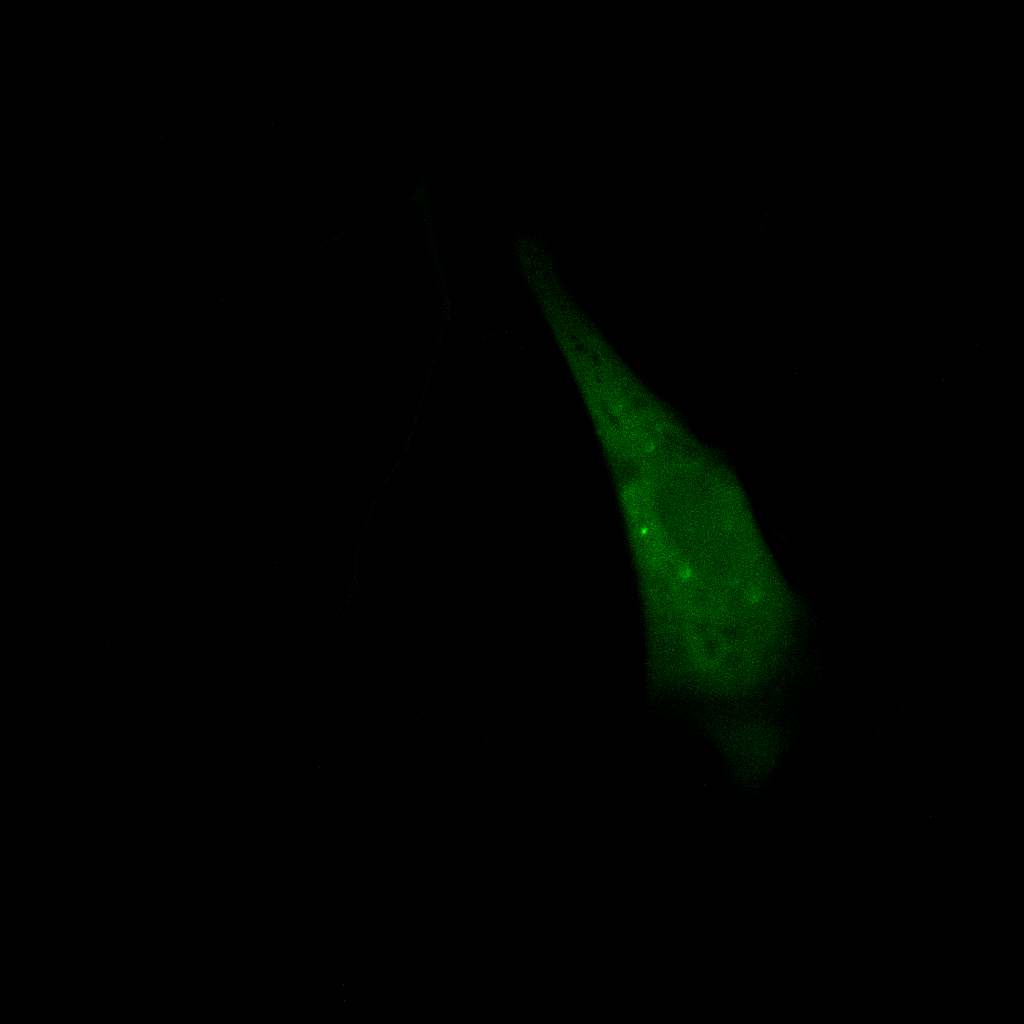


**10 μM**

**Full length figures in figure 5C Supplementary Figure 4**

**
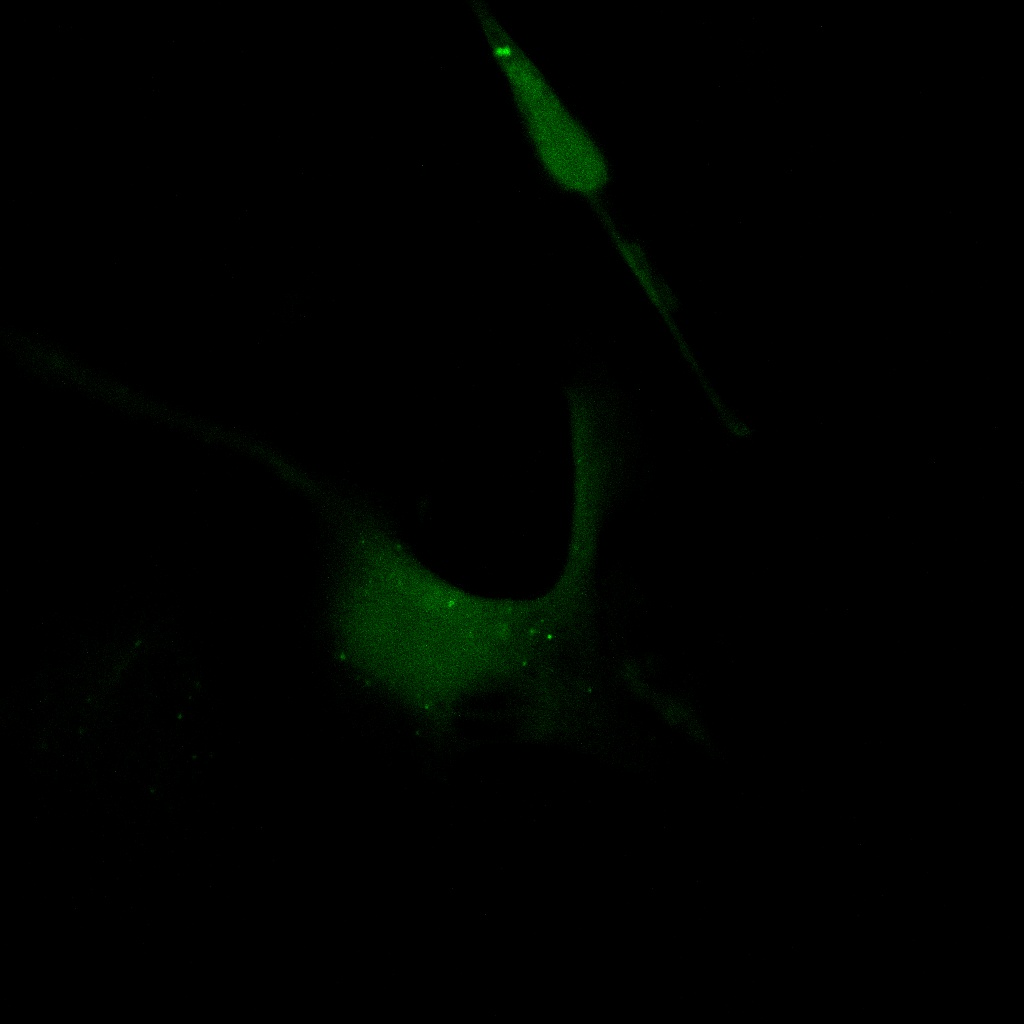

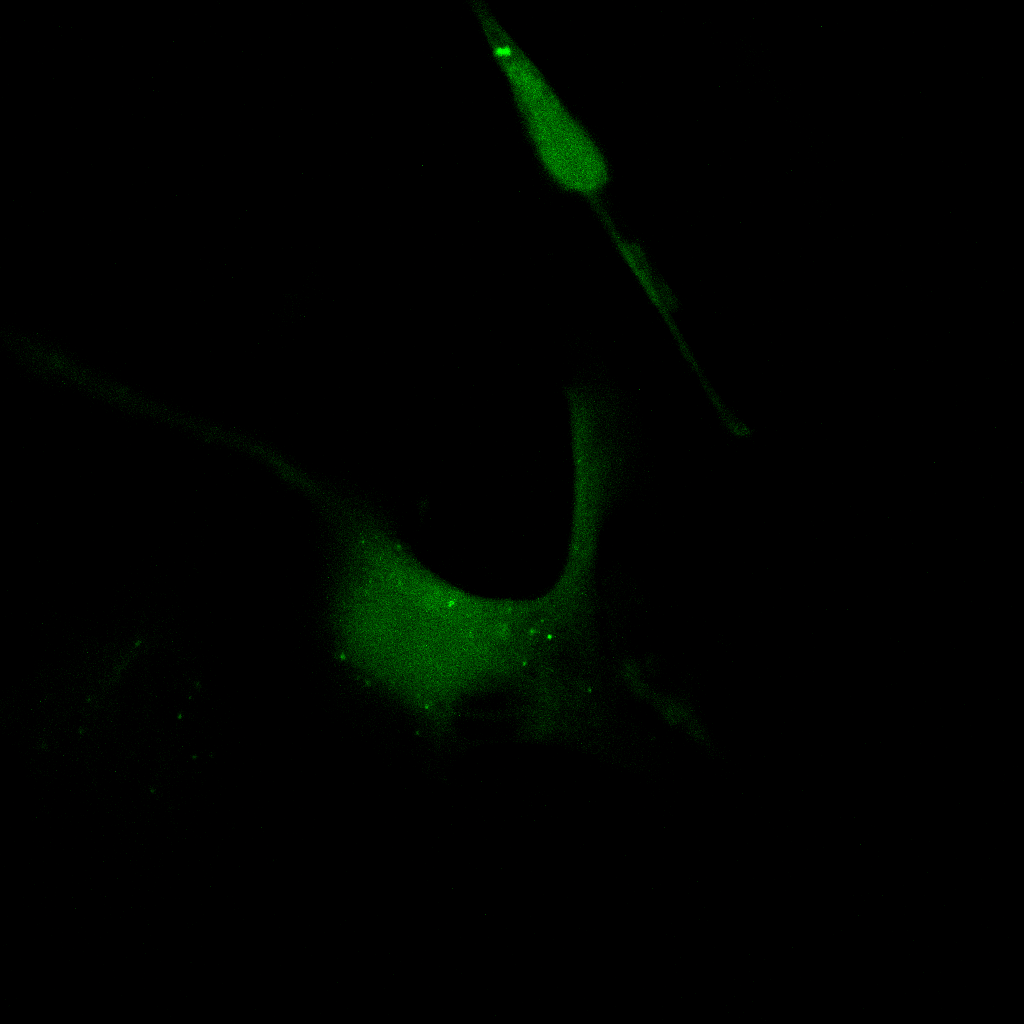
**

**20 μM**

**** **
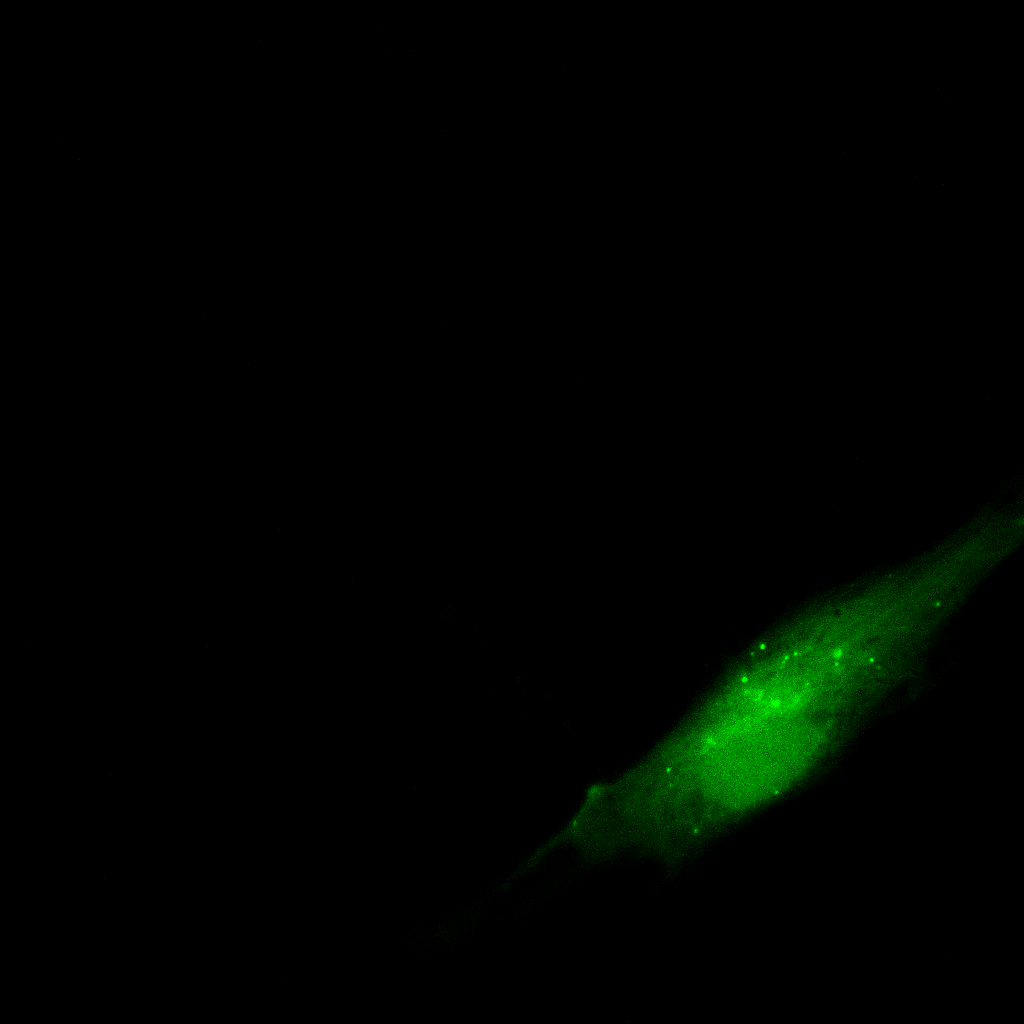
**

**50 μM**

 **
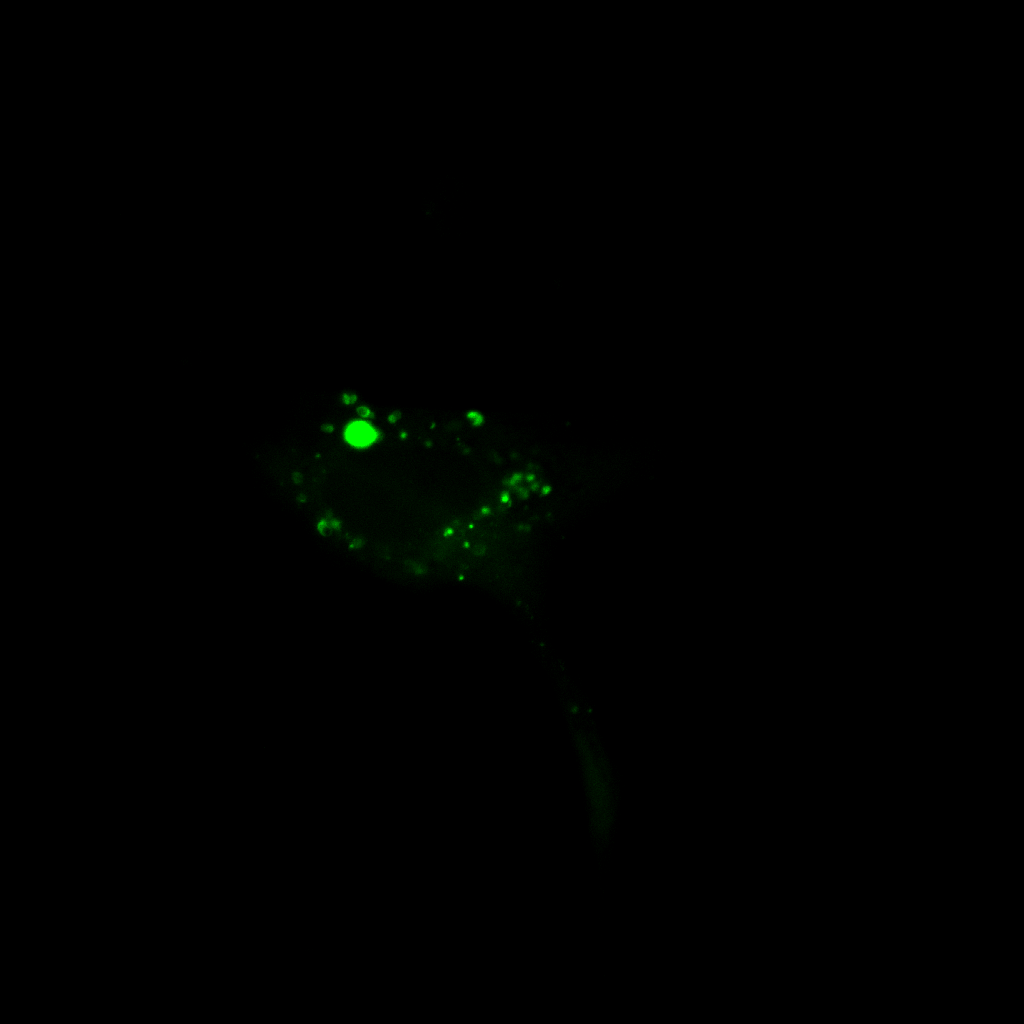
**

Chloroquine 30 μM to induce autophagosomes
